# The modulatory effect of adaptive deep brain stimulation on beta bursts in Parkinson’s disease

**DOI:** 10.1093/brain/awx010

**Published:** 2017-02-13

**Authors:** Gerd Tinkhauser, Alek Pogosyan, Simon Little, Martijn Beudel, Damian M. Herz, Huiling Tan, Peter Brown

**Affiliations:** 1 Medical Research Council Brain Network Dynamics Unit at the University of Oxford, Oxford, UK; 2 Nuffield Department of Clinical Neurosciences, John Radcliffe Hospital, University of Oxford, Oxford, UK; 3 Department of Neurology, Bern University Hospital and University of Bern, Switzerland; 4 Sobell Department of Motor Neuroscience and Movement Disorders, UCL Institute of Neurology, London, UK; 5 University Medical Center Groningen, 9700 RB Groningen, The Netherlands

**Keywords:** Parkinson’s disease, beta oscillations, deep brain stimulation, basal ganglia, closed-loop control

## Abstract

Adaptive deep brain stimulation uses feedback about the state of neural circuits to control stimulation rather than delivering fixed stimulation all the time, as currently performed. In patients with Parkinson’s disease, elevations in beta activity (13–35 Hz) in the subthalamic nucleus have been demonstrated to correlate with clinical impairment and have provided the basis for feedback control in trials of adaptive deep brain stimulation. These pilot studies have suggested that adaptive deep brain stimulation may potentially be more effective, efficient and selective than conventional deep brain stimulation, implying mechanistic differences between the two approaches. Here we test the hypothesis that such differences arise through differential effects on the temporal dynamics of beta activity. The latter is not constantly increased in Parkinson’s disease, but comes in bursts of different durations and amplitudes. We demonstrate that the amplitude of beta activity in the subthalamic nucleus increases in proportion to burst duration, consistent with progressively increasing synchronization. Effective adaptive deep brain stimulation truncated long beta bursts shifting the distribution of burst duration away from long duration with large amplitude towards short duration, lower amplitude bursts. Critically, bursts with shorter duration are negatively and bursts with longer duration positively correlated with the motor impairment off stimulation. Conventional deep brain stimulation did not change the distribution of burst durations. Although both adaptive and conventional deep brain stimulation suppressed mean beta activity amplitude compared to the unstimulated state, this was achieved by a selective effect on burst duration during adaptive deep brain stimulation, whereas conventional deep brain stimulation globally suppressed beta activity. We posit that the relatively selective effect of adaptive deep brain stimulation provides a rationale for why this approach could be more efficacious than conventional continuous deep brain stimulation in the treatment of Parkinson’s disease, and helps inform how adaptive deep brain stimulation might best be delivered.

## Teaser

Pilot studies suggest that adaptive deep brain stimulation (DBS) may be more effective than conventional DBS for the treatment of Parkinson's disease. Tinkhauser *et al*. show that adaptive DBS regulates pathological beta synchronisation in the subthalamic nucleus by selectively limiting long duration beta bursts, which are related to clinical impairment.

## Introduction

Deep brain stimulation (DBS) is a well-established treatment option for advanced Parkinson’s disease (Deuschl *et al.*, 2006; Hariz, 2012). Conventional DBS, as it is currently available, involves the continuous delivery of fixed and high frequency (∼130 Hz) stimulation. Its widespread adoption as a treatment has motivated attempts to further improve the effectiveness, efficiency and therapeutic window of DBS. Presently, research in this regard has focussed on three fields; the exploration of alternative stimulation pulse durations, shapes and patterns (Moro *et al.*, 2002; Reich *et al.*, 2015), the development of stimulation electrodes with the capacity for current steering (Timmermann *et al.*, 2015) and the delivery of closed-loop, adaptive stimulation instead of the current open-loop treatment (Little and Brown, 2012). The increasing evidence that exaggerated oscillatory synchronization in the beta frequency band (13–35 Hz) in Parkinson’s disease can be consistently seen in the local field potential (LFP) picked-up in DBS target structures and that this is correlated with motor impairment in Parkinson’s disease, has qualified it as a potentially suitable feedback signal for adaptive DBS (Abosch *et al.*, 2012; Little and Brown, 2012; Little *et al.*, 2013). In a first proof-of-principle study in patients with Parkinson’s disease, it was shown that using the beta band activity as a feedback signal for unilateral adaptive DBS led to an improvement in contralateral motor performance superior to that achieved with conventional DBS, while battery consumption was reduced by about half (Little *et al.*, 2013). This has been followed by a further patient series confirming the efficacy of bilateral adaptive DBS (Little *et al.*, 2015) and a case report demonstrating a reduction in dyskinesias in Parkinson’s disease with adaptive DBS using a different control algorithm (Rosa *et al.*, 2015). Moreover, in acute trials, it has recently been shown that speech intelligibility is preserved during adaptive DBS, while speech deterioration was observed during conventional DBS (Little *et al.*, 2016).

The working principle of adaptive DBS in the two patient series described above was based on the observation that beta activity in the LFP is not consistently elevated, but comes in bursts. Indeed, even in the physiological state, beta activity in motor cortico-basal ganglia loops comes in bursts, and it has been suggested that changes in beta activity averaged over long periods or around repeated voluntary movements reflect changes in the probability of beta bursts rather than any smooth modulation of beta activity (Feingold *et al.*, 2015). In healthy non-human primates, bursts of beta oscillations are generally only a few cycles long (Murthy and Fetz, 1992, 1996; Feingold *et al.*, 2015). Although burst durations in human basal ganglia (both healthy and in Parkinson’s disease) are not reported in the literature, the exaggerated mean beta activity levels in Parkinson’s disease raise the possibility of more frequent and/or longer bursts in this condition. Indeed, we might also speculate that the correlation between mean beta power and motor impairment, and treatment-induced changes in mean beta power and corresponding changes in motor impairment might actually partly reflect changes in beta burst frequency and/or duration.

During adaptive DBS, as introduced by Little *et al.* (2013), high frequency stimulation switches on solely when the amplitude of beta rises above a preset threshold and stimulation switches off when amplitude falls below this threshold. The presumption is that the elevated beta in these bursts is serving to index key circuit dysfunction at these times. A candidate for this dysfunction is the very synchronization of incoming signals and local neural activity that, through spatiotemporal summation, underlies the beta activity in the LFP, based on the notion that excessive synchronization compromises information coding capacity and circuit performance (Brittain and Brown, 2014).

So what does adaptive DBS do? Does it act by changing the frequency of beta bursts or by changing the distribution of durations of bursts? And should the latter be borne out, why might the duration of beta bursts be critical? Here we assess these questions, and in so doing, provide important insights in to the dynamic nature of the underlying circuit disturbance in Parkinson’s disease and into the optimal control policies needed to interact with these circuits for maximal therapeutic advantage.

## Materials and methods

### Subjects and surgery

We investigated the effect of adaptive DBS compared to no stimulation (noStim) and conventional DBS on bursts of beta activity in 13 patients (16 hemispheres) with advanced Parkinson’s disease undergoing DBS surgery of the subthalamic nucleus (STN) (Table 1). All subjects gave their written informed consent and the local ethics committee approved the study. The average age was 56.8 ± 2.3 years and the preoperative score on the motor section of the Unified Parkinson’s Disease Rating Scale (UPDRS), was 41.5 ± 3.4 OFF medication and 16.7 ± 1.9 ON medication. The mean disease duration at the time of the surgery was 8.2 ± 0.8 years.

**Table 1:** Clinical and stimulation details

Subject	Stim site	Age, yr	Disease duration	Fr range (Hz)	UPDRS ON/OFF levodopa	Principal DBS indication	Stimulation voltage (V, L/R)	Centre
**1**	L, R	41	7	21 ± 3	50/21	Motor fluctuations	3.4/3.3	London
**2**	L, R	66	6	20 ± 3	42/19	Tremor	3.4/3.4	Oxford
**3**	L, R	52	8	20 ± 6	40/11	Tremor	2.5/2.4	London
**4**	R	50	4	18 ± 3	37/17	Motor fluctuations	2.8	London
**5**	R	57	8	19 ± 3	42/29	Motor fluctuations/dyskinesia	2.7	London
**6**	R	67	7	26 ± 3	43/14	Motor fluctuations/dyskinesia	1.8	Oxford
**7**	R	67	14	17–22	63/24	Motor fluctuations	2.4	London
**8**	R	62	10	22 ± 3	20/8	Motor fluctuations/tremor	1.8	London
**9**	L	49	10	17–24	42/6	Tremor	1.6	Oxford
**10**	R	63	3	31 ± 3	18/8	Tremor/ bradykinesia	2.6	Oxford
**11**	L	49	10	17 ± 1	58/23	Motor fluctuations/tremor	2.1	London
**12**	L	59	12	19 ± 3	42/20	Motor fluctuations/tremor/ bradykinesia	2.7	Oxford
**13**	R	57	8	16–20	43/17	Motor fluctuations	2.7	London
**Mean ± SEM**		56.8 ± 2.3	8.2 ± 0.8	20.9 ± 0.95	41.5 ± 3.4/ 16.7 ± 1.9		2.6 ± 0.15	

Stim = stimulation; yr = year; R = right; L = left; SEM = standard error of the mean; Fr = frequency.

Ten of the subjects have been previously reported, seven of them in a unilateral adaptive DBS study (Little *et al.*, 2013) and three of them in a bilateral adaptive DBS study (Little *et al.*, 2015). These previous publications focused on clinical effects and power savings during adaptive DBS. Here we focus on the underlying electrophysiological changes induced by adaptive DBS and how they may relate to clinical changes. Inclusion criteria in the current study were the same as in these previous studies, but subjects also had to demonstrate improvement in motor performance during adaptive DBS compared to no stimulation, as assessed with selected items (20, 22, and 23) of the UPDRS Part III.

### Experiments and recordings

The operative procedure and experimental setting have been described in our previous adaptive DBS studies (Little *et al.*, 2013, 2015). In summary, the LFP recording took place 3–6 days following the implantation of DBS leads (quadripolar macroelectrode, model 3389, Medtronic) and prior to internalization of the wires and connection to the subcutaneous impulse generator. All subjects were withdrawn from dopaminergic medication overnight prior to the recordings. Bipolar recordings were performed from contacts 0–2 and 1–3, band-pass filtered between 3 and 37 Hz and amplified using a 3-stage common mode rejection amplifier. The bipolar contact pair that showed the highest beta band activity (13–35 Hz) was chosen for further recording. The frequency of the peak activity within the beta frequency range was determined and the signal digitally filtered around this peak frequency (Table 1). The filtered LFP signal was rectified and smoothed using a moving average filter of 400-ms duration to produce an online value of beta amplitude. Conventional test stimulation was performed to determine the stimulation voltage that provided the best clinical benefit, without side effects such as paraesthesia. During adaptive DBS, stimulation was only delivered when the beta amplitude crossed a threshold set to deliver stimulation ∼50% of the time. The delay between threshold crossing and onset of stimulation was 30–40 ms. To overcome paraesthesia during the switching on and off of stimulation, stimulation was ramped-up in 250 ms until the target voltage value was reached. Once the stimulation was triggered, it was sustained until beta amplitude fell below threshold again. Stimulation was delivered from the contact in-between the two used for recording, in a monopolar stimulation mode at 130 Hz, with a pulse duration of 100 μs.

### Clinical testing

All patients were clinically assessed during the no stimulation (noStim) session using the UPDRS and patients were not informed about the order of the randomly assigned experimental conditions. Those motor UPDRS items that were consistently tested across all patients were finger tapping (item 23), upper limb rigidity (item 22) and upper limb rest tremor (item 20) so these were therefore totalled for the arm contralateral to recording side and used for further statistical analysis.

Between each condition a 5 min washout period took place. The exact timing of the clinical evaluation has been reported previously (Little *et al.*, 2013, 2015).

### Determination of beta bursts

**Figure 1:**
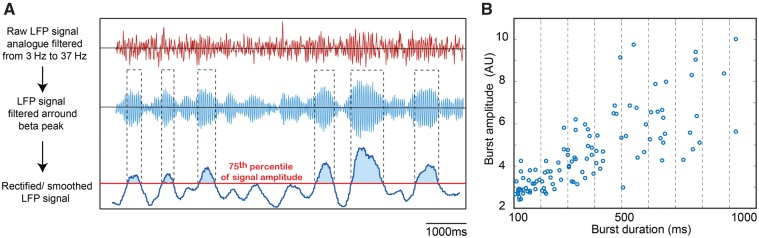
**Steps of burst determination.** (**A**) The analogue LFP signal was filtered around individual beta peak frequency (Table 1). The signal was rectified and smoothed to obtain the envelope of the beta activity. For each condition a threshold was then set at the 75th percentile of the beta amplitude. The onset of a burst was defined as when the rectified signal crossed the threshold amplitude and the end of the burst defined as when the amplitude fell below threshold. (**B**) All bursts with a duration longer than 100 ms were considered. Bursts were further categorized according to their duration into nine time windows (see ‘Materials and methods’ section). Example of burst scatterplot with burst shown up to 1000 ms (Subject 3, left side).

Figure 1A illustrates the processing steps involved in the discrimination of bursts of beta activity. The digitally filtered and the rectified bipolar LFP signal that was generated online as a substrate for thresholding was also recorded for off-line analysis. Such data were first visually inspected and 200 s of artefact-free rectified signal in each condition was selected using Spike2 software (Cambridge Electronic Design).

All further signal processing and data analysis steps were performed using Matlab (version R 2015b; MathWorks, Natick, MA). Data were downsampled to 200 Hz. To adjust for potential baseline shifts, we performed a direct current (DC) correction (20 s time constant) of the selected rectified signal.

Having focused on the variance in the beta signal by the above processing we compared noStim, adaptive and conventional DBS conditions by using a common threshold definition for beta bursts across conditions. The duration of the beta bursts was determined from the time points at which the DC corrected, smoothed, rectified and filtered beta signal exceeded a given threshold amplitude and then fell below this threshold. Thresholds were defined in terms of percentiles of the signal amplitude distribution. However, as the precise amplitudes of percentile-defined thresholds could vary between conditions, the applied threshold was set as the average of the amplitudes corresponding to the selected percentile, and the same threshold applied to all conditions for a given hemisphere. Thus when the text refers to, for example, thresholding according to the 75th percentile, the same threshold equivalent to the mean of the 75th percentiles across conditions, is applied to each condition. The same mean percentile threshold amplitude was applied to all the different conditions to allow for the potentially greater variance of the signal during adaptive DBS due to the mixing of on and off stimulation periods. Had we not done this, a given percentile threshold may have been of higher amplitude in adaptive DBS than in conventional DBS, and therefore burst duration potentially relatively underestimated in adaptive DBS. However, common thresholds were not applied when comparing burst amplitudes between conditions (see below). Here bursts were classified according to the condition-specific amplitude percentile threshold (threshold was not averaged across conditions). This was necessary so as to not overestimate burst amplitude during adaptive DBS due to different noise floor levels between conditions.

The selection of a given percentile amplitude threshold according to the distribution of signal dynamics is somewhat arbitrary. Empirical findings indicate that a minimum beta elevation of ∼50% is sufficient to have a satisfactory clinical effect, but could not exclude similar effects with higher thresholds (Little *et al.*, 2013, 2015). Accordingly, we looked for a consistent change in distributions of beta bursts defined by a range of different percentile thresholds above this minimum (55, 60, 65, 70, 75, 80, 85, 90th percentiles) and integrated these in our results. In addition, in the results we present the findings for the 75th percentile threshold in more detail, as exemplar.

The distribution of burst durations was considered by categorizing them into nine time windows of 100 ms starting from 100 ms to >900 ms in duration (Fig. 1B). Note that the last time window (>900 ms) includes bursts with a broader range of duration. This was necessary as longer bursts became progressively less frequent and in this way we ensured sufficient burst numbers in each window. We did not consider bursts with durations shorter then 100 ms (less that about two cycles in duration) to limit the contribution of spontaneous fluctuations in amplitude due to noise.

As noted above, the rectified signal was filtered around the individual beta peak frequency. To investigate if small shifts in the beta frequency peak within a subject or condition could affect the results of the burst distribution, the described algorithm to derive beta bursts was also applied on the Hilbert envelope of the broadly filtered signal (13–30 Hz) and key analyses were repeated (see below).

For eight subjects (Cases 6–13) that were drawn from the unilateral adaptive DBS study a random DBS condition was also available (Little *et al.*, 2013). Random periods of DBS were delivered with a duration distribution and frequency that was similar to that delivered during adaptive DBS, except that in the latter case stimulation periods were triggered by elevated beta activity in the STN region LFP. Beta bursts during random DBS were derived with the same above-described algorithm.

The motivation for focusing on beta burst duration was that this is the obvious factor that adaptive DBS may change. Thereby we hoped to identify if and how any change in the distribution of beta burst duration might impact on the clinical state. Previous studies have shown that conventional DBS suppresses the level of beta activity relative to the unstimulated OFF medication state when activity is averaged over long periods (Eusebio *et al.*, 2011; Quinn *et al.*, 2015; Neumann *et al.*, 2016). Whether adaptive DBS does the same has not been explicitly reported (Little *et al.*, 2013, 2015; Rosa *et al.*, 2015), perhaps because, in our experience, this form of stimulation can be associated with a change in the effective noise floor of the amplifiers used. A similar problem can also be seen with continuous high frequency stimulation, due, in part to an increase in 1/f noise during stimulation (Afshar *et al.*, 2012; Stanslaski *et al.*, 2012). However, this problem may be exacerbated in the case of adaptive DBS because stimulation is intermittent. In particular, in some patients we observed a low frequency component to the signal at the onset and offset of each burst of stimulation, which may possibly reflect a capacitance change at the brain electrode interface or an amplifier transient. It was not observed at the onset and offset of conventional DBS, but this is most likely because conventional DBS was ramped up over several seconds.

In the current study, irrespective of overall beta levels, we explore whether there is any change in the distribution of the beta bursts; in effect we focus on an aspect of the beta signal variance rather than on its mean. This was achieved by DC correction of the rectified smoothed signal. The 20 s time constant of this correction made allowances for any slow shifts in the mean baseline level of beta, thereby further focusing our analysis on the more dynamic elements of the signal variance, the beta bursts. A practical benefit of the above approach is that it also limited any impact of changes in the effective noise floor of the amplifier upon which biological changes in LFP activity are superimposed. This noise floor may potentially vary between adaptive and conventional DBS, particularly at low frequencies, as discussed above.

An alternative approach to our DC correction method might have been to normalize the stimulation conditions by the noStim data. However, this approach has the disadvantage that the results may have been affected by any change in the effective noise floor of the amplifier in and between stimulation conditions. It would also fail to limit the effect of any slow shifts in the mean baseline level of beta during stimulation. Note that in practice, there was no difference in the mean amplitude of the threshold level between conditions across subjects in our data (see ‘Results’ section).

### Data analysis and statistics

The burst results were derived at the level of each hemisphere. To investigate the distribution of bursts with different durations between the conditions, we performed a two-way repeated measures ANOVA with a 9 × 3 design (nine time windows, three conditions). Additionally, for each hemisphere the total number of bursts was calculated and compared using a one-way repeated measures ANOVA. The burst distribution was further analysed as a dichotomized distribution consisting of short bursts (100 ms to 600 ms) and long bursts (>600 ms) and two-way repeated measures ANOVA repeated on these data with a 2 × 3 design (two time windows, three conditions). In a second step the burst distribution was replaced with the total time spent during short and long bursts and the same analyses applied. To investigate the total number of bursts and total time spent in short and long bursts, we averaged the dichotomized burst distributions derived from each percentile threshold (percentage normalization to the sum of the number or total duration of all bursts across time windows) and performed a two-way repeated measures ANOVA with a 2 × 3 design (two time windows, three conditions). Significant main effects and interactions were further investigated with *post hoc* tests.

The mean amplitude of the bursts with different duration time windows and different stimulation conditions was also calculated. The relation between burst amplitude and burst duration was assessed by applying Spearman bivariate correlation (rs = Spearman’s rho), separately at the group level and within hemispheres. The same bivariate correlation was repeated for each threshold, and the average r’s for each condition, as well as the number of significant correlations out of 16 cases, are reported. To investigate the effect of stimulation condition on the overall burst amplitude, we averaged the condition-specific amplitude across all duration time windows for each hemisphere. We then compared the means by applying a one-way repeated measures ANOVA. For the comparison of the amplitude suppression across the different thresholds, we again averaged the condition-specific amplitude across all duration time windows for each hemisphere, followed by a percentage normalization (to the sum amplitude across the conditions) to permit comparison across thresholds.

To explore the effect of stimulation on the integrated burst amplitude with different time windows, we separately multiplied the window-specific averaged amplitude with the corresponding burst probability within the 200 s analysis epoch for noStim, adaptive and conventional DBS. Thereafter, the time window-specific values were normalized in order that the sum of the integrated amplitude of all the time windows per condition was equal to 100%. To compare the means, we applied a two-way repeated measures ANOVA. For the comparison across threshold we averaged the normalized integrated data from each threshold and repeated the two-way repeated measures ANOVA.

For the noStim condition we investigated the relationship between burst characteristics and motor performance by applying Pearson's correlation analyses. We used the percentage amount of bursts for each time window and correlated them with the clinical impairment, to determine the overall relationship between burst duration and motor performance (see ‘Results’ section). The motor performance was given by the sum of UPDRS Part III items 20, 22 and 23 contralateral to the stimulated side and bursts as percentage of short bursts and long bursts relative to all the bursts from 100 ms to >900 ms.

Statistical analyses were performed using IBM SPSS Statistics Version 23. All data are presented as means ± SEM, unless otherwise stated. The assumption of a normal distribution was tested by visual inspection of the QQ-plots. For all repeated measures ANOVAs, if Mauchly’s test indicated that the assumption of sphericity was violated, Greenhouse-Geisser corrections were applied. In this case, corrected *F* and *P-*values with corrected degrees of freedom were reported. *Post hoc* paired *t*-tests were Bonferroni corrected. All reported *P*-values from the correlation analyses have been corrected for multiple comparisons using the false discovery rate procedure.

## Results

### Change in distribution of burst durations during adaptive deep brain stimulation

**Figure 2:**
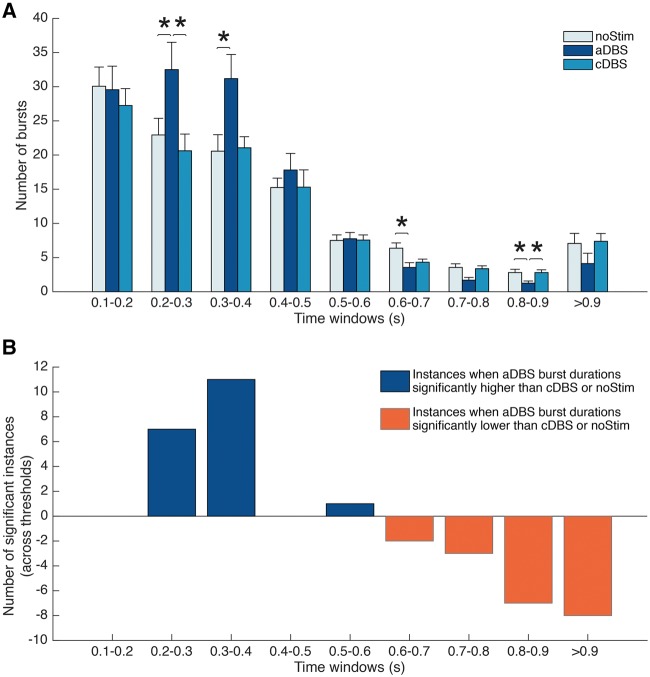
**Beta burst distributions during noStim, adaptive and conventional DBS.** (**A**) Number of bursts of different durations during noStim, adaptive and conventional DBS, where bursts are defined as periods of beta activity that exceed the 75th percentile for longer than 100 ms. For adaptive DBS the number of shorter bursts (200–400 ms) is higher and the number of longer bursts (600–700 and 800–900 ms) is lower compared to noStim. The comparison between adaptive and conventional DBS similarly shows a higher number of short bursts (300–400 ms) and a lower number of long bursts (800–900 ms) during adaptive DBS. The number of bursts of different durations did not differ between noStim and conventional DBS. Values are represented as mean + SEM; ^*^P < 0.05. (**B**) Summarizes the data from different thresholds by showing how often the number of bursts of a given time window during adaptive DBS significantly differed from those of the same time window during conventional DBS or noStim (Supplementary Fig. 1). aDBS = adaptive DBS; cDBS = conventional DBS.

Figure 2A illustrates the number of bursts within the 200 s analysis epoch across different durations and conditions when bursts were defined as beta amplitude exceeding the 75th percentile for a minimum of 100 ms. A repeated measures ANOVA showed the following effects on the number of bursts in the 200 s recording: stimulation condition *F*(2,30) = 2.568, *P* = 0.093, burst duration *F*(2.6,39.4) = 55.139, *P* < 0.001 and interaction between stimulation condition and burst duration *F*(4.3,64.7) = 4.754, *P* = 0.002. For the *post hoc* comparison between adaptive DBS and noStim we found that the number of short bursts (200–300 ms and 300–400 ms) was higher during adaptive DBS compared to noStim [*t*(15) = 2.750, *P* = 0.045; *t*(15) = 3.126, *P* = 0.049]. The opposite was observed for the long bursts (600–700 ms and 800–900 ms) where the number of bursts during adaptive DBS was lower compared to noStim [*t*(15) = −2.883, *P* = 0.034; *t*(15) = −2.707, *P* = 0.006]. Between adaptive and conventional DBS we found a similar difference in the burst distribution pattern. The number of short bursts (200–300 ms) was higher during adaptive DBS compared to conventional DBS [*t*(15) = 3.250, *P* = 0.016], while the number of long bursts (800–900 ms) was lower in adaptive DBS compared to conventional DBS [*t*(15) = −4.753, *P* = 0.001].

However, the details of distributions of beta bursts will depend on the criteria used to define an amplitude elevation. Empirical findings indicate that stimulating once a minimum beta elevation of ∼50% is sufficient to have a satisfactory clinical effect, but could not exclude similar effects with higher thresholds (Little *et al.*, 2013, 2015). Accordingly we looked for a consistent change in distributions of beta bursts defined by different thresholds above this minimum. The number of beta bursts of different duration per 200 s in a given stimulation condition for different thresholds over 55–90% are shown in Supplementary Fig. 1A–H.

**Figure 3:**
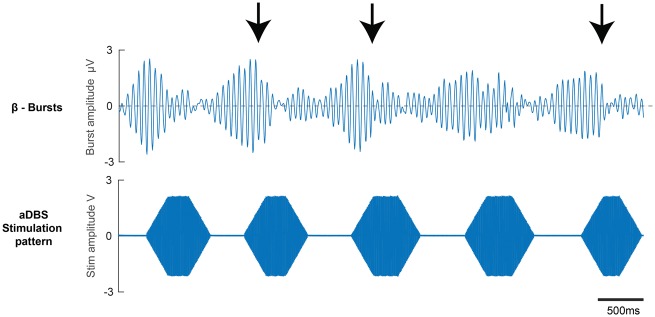
**Burst trimming effects of adaptive DBS illustrated on a series of beta bursts.** Subject 11, left side. The *top* row shows a consecutive sequence of beta bursts observed during adaptive DBS. The *bottom* row shows the stimulation pattern as induced by the corresponding bursts above. Bursts appear terminated by the triggered stimulation and bursts with black arrows do end relatively abruptly, coinciding with the ramp-up of stimulation. aDBS = adaptive DBS.

Figure 2B summarizes the data from different thresholds by showing how often the number of bursts of a given duration during adaptive DBS significantly differed from those of the same duration during noStim or conventional DBS. The figure suggests that beta bursts shorter than 600 ms tended to increase in their frequency during adaptive DBS, whereas beta bursts longer than 600 ms tended to reduce in their frequency during adaptive DBS. By way of comparison, only one significant difference in the distribution of burst durations was found between noStim and conventional DBS in a similar comparison across different thresholds. The selective suppression of longer bursts during adaptive DBS is in line with the delivery of adaptive DBS, which involves delays due to signal filtering and smoothing (using a 400 ms moving window average), and due to the ramping nature of stimulation once triggered so that it takes a further 250 ms before clinically effective voltage levels are reached. Figure 3 shows some examples of asymmetric longer beta bursts consistent with abrupt termination after ∼600 ms by the triggered high frequency DBS.

**Figure 4:**
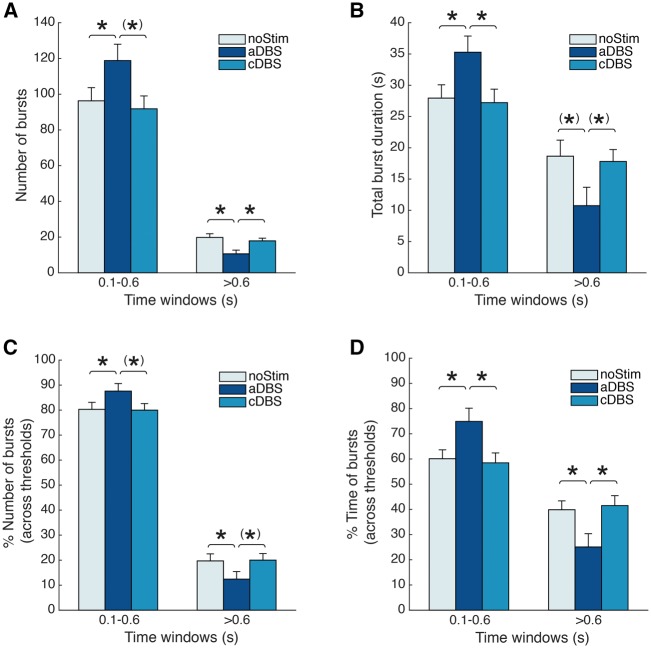
**Dichotomized distribution for short and long bursts during noStim, adaptive and conventional DBS.** (**A**) The number of normalized bursts (categorized into short bursts: 100–600 ms and long bursts: >600 ms) for the 75% threshold, which confirms a similar pattern with a higher number of short bursts in adaptive DBS (compared to noStim) and a lower number of long bursts in adaptive DBS (compared to conventional DBS and noStim). (**B**) The total burst duration of the normalized bursts for the 75% threshold. This confirms a higher total time of short bursts for adaptive DBS (compared to noStim and conventional DBS) and a lower total time of long bursts in adaptive DBS (compared to noStim and conventional DBS). (**C**) The burst distribution as percentage normalized bursts for noStim, adaptive and conventional DBS across the various thresholds (see also Supplementary Fig. 1). The corresponding repeated measures ANOVA again confirmed an interaction between stimulation condition and burst duration [*F*(2,30) = 6.627, *P* = 0.004], the corresponding *post hoc* tests indicate a higher amount of shorter bursts in adaptive DBS compared to noStim [*t*(15) = 3.056, *P* = 0.024] and conventional DBS [*t*(15) = 2.643, *P* = 0.055], while the number of longer bursts is lower in adaptive DBS compared to noStim [*t*(15) = − 3.056, *P* = 0.024] and conventional DBS [*t*(15) = − 2.643, *P* = 0.055]. No difference in the distribution for short and long bursts was found between noStim and conventional DBS [*t*(15) = 0.188, *P* = 1; *t*(15) = − 0.188,*P* = 1]. (**D**) The percentage distribution of the total burst duration for the normalized bursts across the various thresholds. The repeated measures ANOVA confirmed again a significant interaction between condition and duration of bursts [*F*(1.4,21.6) = 9.648, *P* = 0.002]. The *post hoc* tests showed a longer total duration for short bursts in adaptive DBS compared to noStim [*t*(15) = 3.303, *P* = 0.014] and conventional DBS [*t*(15) = 3.336, *P* = 0.014], while the total duration for long bursts was reduced in adaptive DBS compared to noStim [*t*(15) = − 3.303, *P* = 0.014] and conventional DBS [*t*(15) = − 3.336, *P* = 0.014]. Again no difference in the total duration for short and long bursts was found between noStim and conventional DBS [*t*(15) = 0.641, *P* = 1; *t*(15) = − 0.641, *P* = 1]. Values are represented as mean + SEM; ^*^P < 0.05. Asterisks in brackets: *P*-value significant before correction for multiple comparisons (Bonferroni) only.

Although there was a trend for adaptive DBS to have a higher total number of bursts (regardless of their duration over 100 ms) than conventional DBS or noStim over the different thresholds, this was not significant in an ANOVA with threshold and stimulation as main effects (Supplementary Fig. 2). However, this could hide a different distribution of bursts of different duration in the three conditions. Consequently, and in line with the above findings, we divided burst durations according to whether they were less than or greater than 600 ms in duration. Figure 4A illustrates the results for the 75% threshold. The repeated measures ANOVA on this dichotomized burst distribution defined at this threshold showed a significant main effect on the interaction between stimulation condition and burst duration [*F*(1.5,21.8) = 9.111, *P* = 0.003]. Consistent with the previous results, the *post hoc* tests showed an increased number of short bursts in adaptive DBS compared to noStim [*t*(15) = 2.976, *P* = 0.028] and conventional DBS (*t* = 2.647, *P* = 0.055), although the latter did not quite reach statistical significance after correction for multiple comparisons (corrected *P-*values given here and elsewhere in the text). The opposite was true for long bursts, here the number decreased during adaptive DBS compared to both noStim [*t*(15) = −2.979, *P* = 0.028] and conventional DBS [*t*(15) = −3.385, *P* = 0.012]. No difference was found between noStim and conventional DBS, for either short bursts [*t*(15) = 0.596, *P* = 1] or long bursts [*t*(15) = 0.792, *P* = 1].

The same pattern was seen using the 75% threshold definition if the total number of bursts was replaced by the total time spent in each of the two burst duration windows (Fig. 4B). The time spent in short bursts in noStim totalled on average 27.9 s (ranging from 5.5 s to 37.6 s), in adaptive DBS 35.3 s (ranging from 8.7 s to 46.1 s) and in conventional DBS 27.2 s (ranging from 10.5 s to 39.0 s). For long bursts the average total duration during noStim was 18.7 s (ranging from 4.9 s to 48.3 s), during adaptive DBS 10.7 s (ranging from 0.61 s to 40.7 s) and during conventional DBS 17.8 s (ranging from 6.5 s to 29.6 s). The repeated measures ANOVA again confirmed a significant interaction between stimulation condition and burst duration [*F*(2,30) = 9.525, *P* = 0.001]. *Post hoc* tests revealed a longer total burst duration for short bursts in adaptive DBS compared to noStim [*t*(15) = 3.388, *P* = 0.012] and conventional DBS [*t*(15) = 2.707, *P* = 0.049]. The opposite was found for the total duration of long bursts, which was lower in adaptive DBS compared to both noStim [*t*(15) = −2.416, *P* = 0.087] and conventional DBS [*t*(15) = −2.472, *P* = 0.078], although the corrected *P*-values of the latter two comparisons did not reach statistical significance. Again no difference was found between noStim and conventional DBS, for either short bursts [*t*(15) = 0.296, *P* = 1] or long bursts [*t*(15) = 0.323, *P* = 1].

How did the dichotomized burst distribution look if, instead of considering the representative 75th percentile threshold only, we also included the burst distribution from lower and higher thresholds? As the total number of bursts depended on the selected threshold, we expressed the number of bursts of short or long duration as a % of the total number of bursts for each threshold to avoid group average data being dominated by the results from lower thresholds. Figure 4C shows the result of this analysis for the dichotomized burst durations in each stimulation condition averaged across hemispheres and thresholds. The corresponding repeated measures ANOVA confirmed an interaction between stimulation condition and burst duration [*F*(2,30) = 6.627, *P* = 0.004], due to more short bursts and fewer long bursts during adaptive DBS than noStim or conventional DBS. The result was similar if the per cent of the total number of bursts was replaced by the time spent in each burst window as a per cent of the total duration of all bursts (Fig. 4D). The corresponding repeated measures ANOVA again confirmed an interaction between stimulation condition and burst duration [*F*(1.4,21.6) = 9.648, *P* = 0.002]. Corresponding *post hoc* tests for the dichotomized distribution across thresholds are illustrated in Fig. 4. Thus adaptive DBS caused a shift from longer to shorter bursts relative to noStim and conventional DBS, despite the lack of change in the overall number of bursts in any condition. The balance of short and long bursts did not change during conventional DBS relative to that during noStim.

### Change in burst distribution during adaptive deep brain stimulation is not altered if filtering of beta bursts is relaxed

As part of the signal processing to derive beta bursts we digitally filtered the STN region LFP around the frequency of the peak activity in the beta band (Table 1; mean pass band centred on peak was ∼ 21 ± 5 Hz wide). This raises the possibility that, rather than a genuine shift in beta bursts from long to short duration during adaptive DBS, there may have been a frequency shift brought about by adaptive DBS that meant that burst frequency during long duration bursts wandered out of the relatively narrow pass band used in the analysis. To militate against this possibility, we therefore repeated the signal processing, but with a much broader beta pass band from 13–30 Hz (width 18 Hz). The number of bursts and the corresponding total burst duration for the representative threshold of 75% are shown in Supplementary Fig. 3A and B. ANOVAs based on these data confirmed an interaction between factors stimulation condition and burst duration (Supplementary Fig. 3). These data suggest that shifts in burst duration could not be fully explained by shifts in burst frequency during DBS.

### Burst distribution during adaptive deep brain stimulation differs to that during random stimulation

Could the reduction in long duration bursts during adaptive DBS be somehow related to stimulation artefact and amplifier noise floors, which potentially differ between adaptive and conventional DBS? Most likely, any potential contamination of bursts by stimulation-related artefact would have only served to increase the number of beta bursts with durations >600 ms during adaptive DBS. However, to address this question more directly, we contrasted data from those eight patients who had both trials of unilateral adaptive DBS and random DBS drawn from Little *et al.* (2013). In the latter study, random periods of DBS were delivered with a duration distribution and frequency that was similar to that delivered during adaptive DBS, except that in the latter case stimulation periods were triggered by elevated beta activity in the STN region LFP. Adaptive DBS still had fewer long beta bursts than random DBS when using a common amplitude threshold (mean of 75% percentiles) to define bursts across the two conditions [number of bursts >600 ms, 12.8 ± 3.2 and 16.9 ± 3.2 over 200 s, respectively; *t*-test, *t*(7) = 2.601, *P* = 0.035].

### Short and long duration bursts comprise only a fraction of the subthalamic nucleus region local field potential

The total duration of time spent out of the 200 s recordings in either short or long bursts, with these defined as less than or greater than 600 ms in duration, clearly depended on the amplitude threshold used. Supplementary Fig. 4 illustrates this across the different thresholds. However, the summed duration of short burst durations was greatest, and that of long burst durations shortest for adaptive DBS (seen for the 60 to 90 percentile thresholds) (Supplementary Fig. 4).

### Burst amplitude increases with burst duration

**Figure 5:**
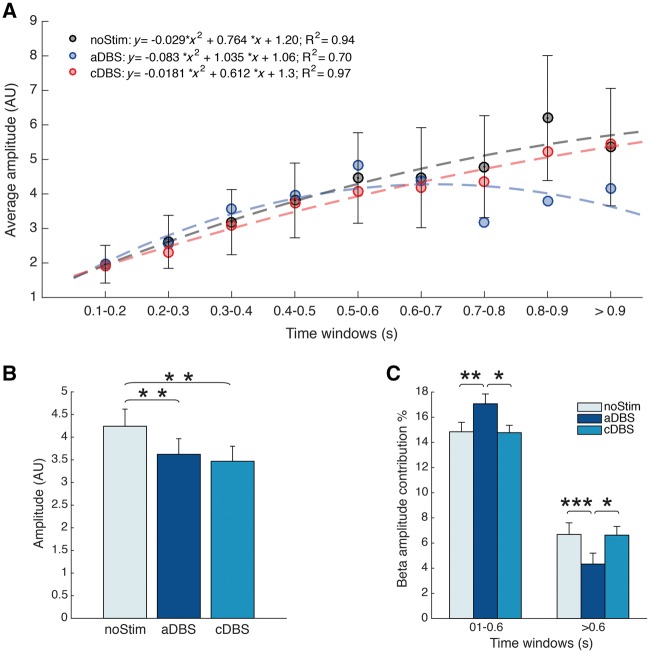
**Relationship between burst duration and burst amplitude for the representative 75% threshold.** (**A**) The mean amplitudes for different durations for noStim, adaptive and conventional DBS. SEMs are shown for noStim only. The strong positive correlation indicates the longer the burst duration, the higher the burst amplitude. A slight flattening of this relation can be seen during adaptive DBS at longer burst durations. A second order polynomial was fitted to the data of the three conditions (see equations). Within subjects, a significant correlation could be found for almost all the hemispheres and conditions (noStim 14/16, adaptive DBS 11/16, conventional DBS 13/16). The results of the correlation analyses between burst duration and burst amplitude across all the thresholds are shown in Supplementary Fig. 5. (**B**) Mean burst amplitude for noStim, adaptive and conventional DBS averaged across hemispheres and time windows. Both adaptive DBS and conventional DBS show a significant reduction in beta amplitude compared to noStim. However, no difference was found between adaptive and conventional DBS. (**C**) Integrated burst amplitude (normalized to 100%, which corresponds to total integrated amplitude summed across all time windows) for short bursts (100–600 ms) and long bursts (>600 ms). Stimulation conditions show significantly different amplitude effects when burst duration is considered. Adaptive DBS has a higher integrated amplitude in shorter bursts, while conventional DBS and noStim have a higher integrated amplitude in longer bursts. Supplementary Fig. 7 illustrates mean amplitude as well as the integrated amplitude across the different thresholds. Values are represented as mean + SEM; ^*^P < 0.05, ^**^P < 0.01, ^***^P < 0.001. aDBS = adaptive DBS; cDBS = conventional DBS.

In summary, adaptive DBS changed the distribution of beta bursts so that there were fewer long bursts, and more short bursts in adaptive DBS than in conventional DBS or noStim. This was also reflected in the accumulated duration of such bursts over the 200 s recording. But why should the frequency of long bursts matter? This may be explained by a significant positive correlation between burst duration and burst amplitude. Figure 5A illustrates the relationship at a group level across conditions using the same representative threshold of 75% as elsewhere. A significant positive correlation was consistently present within hemispheres for noStim (14/16 hemispheres significant, mean rs = 0.8350 ± 0.0393), adaptive DBS (11/16 significant, rs = 0.8144 ± 0.0281) and conventional DBS (13/16 significant, rs = 0.8451 ± 0.0247), and when repeated for other thresholds between 55 and 90% (Supplementary Fig. 5A and B). Thus bursts with longer duration had higher average amplitudes. Supplementary Fig. 6 shows representative time frequency spectra of the three conditions and demonstrates that high amplitude bursts were reduced during adaptive DBS relative to conventional DBS, although beta activity was generally suppressed in this latter condition.

### Burst amplitude averaged over all burst durations is suppressed

Hitherto we have used a common threshold across stimulation conditions, defined by the average of the amplitudes corresponding to the specified percentile threshold across these conditions. This was to avoid the potential for underestimating burst duration during adaptive DBS, which may have had a higher variance given the mixing of on and off stimulation periods. However, this approach might lead to an overestimation of burst amplitude during adaptive DBS. To militate this possibility, we compared the burst amplitude averaged across hemispheres and time windows separately for all three conditions using the condition-specific threshold of 75%. Figure 5B illustrates for all three conditions, burst amplitude averaged across hemispheres and time windows. There was a significant main effect for condition in the one-way repeated measures ANOVA [*F*(2,16) = 19.709, *P* < 0.001]. *Post hoc* tests confirmed a higher amplitude in noStim compared to both adaptive DBS [*t*(8) = 4.4597, *P* = 0.005] and conventional DBS [*t*(8) = 6.611, *P* = 0.001]. The mean amplitude for conventional DBS was not different to that for adaptive DBS [*t*(8) = 1.109, *P* = 0.899]. Thus, when averaged across all durations, bursts did not differ in amplitude between adaptive and conventional DBS, although both differed from the amplitude of bursts in the unstimulated case. The findings were similar across different threshold definitions of bursts (Supplementary Fig. 7A and B). The comparable amplitude suppression during adaptive and conventional DBS, as illustrated in Fig. 5B, is most likely explained by the fact that conventional DBS reduced the amplitudes of both short and long bursts, whereas the adaptive DBS algorithm used only reduced the long bursts, which also have higher amplitudes. This effect of adaptive DBS was partially offset by the increase in the numbers of lower amplitude, short bursts during adaptive DBS, so that overall, mean burst amplitude averaged across hemispheres and time windows was similarly suppressed during adaptive and conventional DBS.

### Similar average burst amplitude in adaptive and conventional deep brain stimulation belies a shift in contribution of short and long bursts

Figure 2A and B confirms that the distribution of short and long bursts changes in adaptive DBS as compared to both noStim and conventional DBS. What happens if both mean amplitude of different burst durations and their frequency are simultaneously considered? Figure 5C illustrates the integrated and normalized amplitude of short and long bursts for the representative threshold of 75%. In a two-way repeated ANOVA we found a significant main effect for the interaction between condition and burst duration [*F*(1.3,20.2) = 10.682, *P* = 0.002]. *Post hoc* tests showed that the percentage of integrated amplitude of short bursts (% of the sum of the integrated amplitude across all the time windows) was higher in adaptive DBS compared to noStim [*t*(15) = 4.747, *P* = 0.001] and conventional DBS [*t*(15) = 3.310, *P* = 0.014]. The opposite was true for longer bursts, where the percentage of integrated amplitude from longer bursts in noStim and conventional DBS was higher than in adaptive DBS [*t*(15) = −5.019, *P* < 0.001; *t*(15) = −3.264, *P* = 0.016]. No difference was found between noStim and conventional DBS, either for short bursts [*t*(15) = 0.761, *P* = 1] or for longer bursts [*t*(15) = − 0.647, *P* = 1]. Accordingly, the integrated amplitude for bursts of different duration was increased for short bursts and reduced for long bursts in adaptive DBS. This further underscores that, although both adaptive and conventional DBS have a lower amplitude compared to noStim, bursts of short and long duration make different contributions to the total amplitude in the different conditions. The findings were similar across different threshold definitions of bursts (Supplementary Fig. 7C).

### Clinical correlation

**Figure 6:**
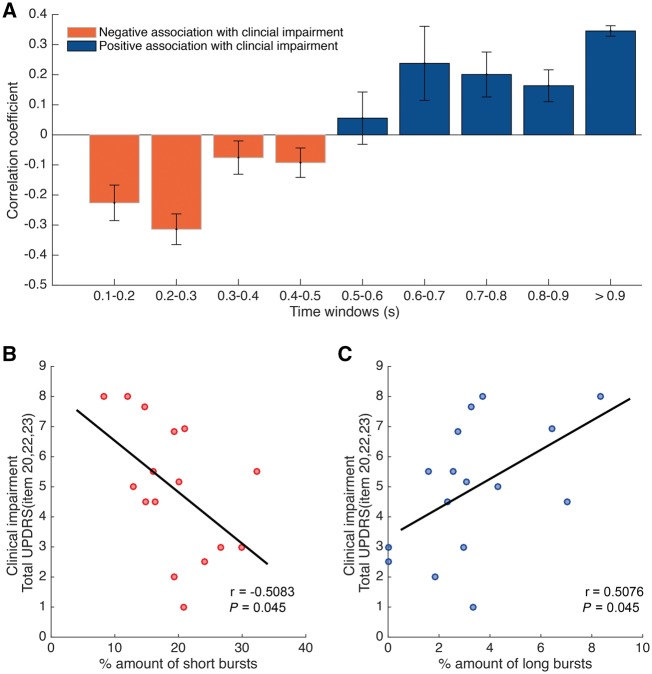
**Clinical correlation between burst duration and clinical impairment.** (**A**) Pearson’s correlations between clinical impairment and the percentage amount of bursts during different burst time windows across the various thresholds. These show that shorter bursts tend to be negatively correlated with clinical impairment and longer bursts tend to be positively correlated with clinical impairment. (**B**) Example scatter plot of percentage amount of short bursts (of 200–300 ms duration) and clinical impairment (UPDRS Part III items 20, 22 and 23 contralateral to the recording side). (**C**) Example scatter plot of percentage amount of long bursts (700–800 ms) and clinical impairment. **B** and **C** are data for representative threshold (75th percentile).

Given the differences seen at the level of burst duration we considered whether the distribution of burst durations was linked to Parkinsonian impairment. To investigate whether bursts of different durations might be correlated with clinical impairment and to confirm how consistent this might be across different thresholds, we correlated all burst time windows from all different thresholds with the clinical impairment (Fig. 6A). Bursts of a shorter duration tended to be negatively correlated with the clinical impairment, whereas the opposite was true for longer bursts. Example scatter plots are shown in Fig. 6B and C. Note that although these scatter plots show significant correlations between burst duration and clinical impairment there was no such correlation between burst duration and average rectified amplitude (r = 0.22, *P* = 0.48 and r = −0.19, *P* = 0.48, respectively). The above clinical correlations were similar when taking the integrated and normalized amplitude of bursts rather than the number of bursts (Supplementary Fig. 8).

## Discussion

**Figure 7:**
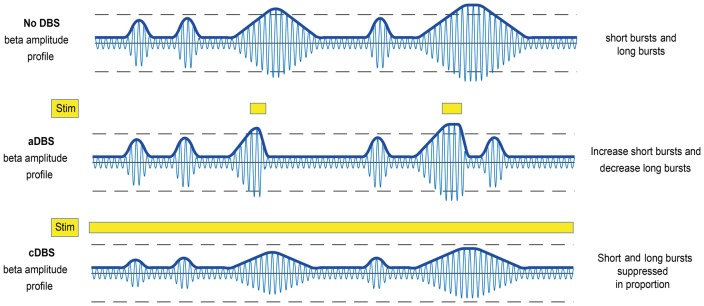
**Summary schematic.** Beta amplitude profile shown during noStim, adaptive and conventional DBS. Without DBS both short and long beta bursts occur. During adaptive DBS the longer bursts are trimmed. This in turn affords more space for shorter bursts to occur. During conventional DBS the distribution of short and long bursts does not change, but overall beta is still suppressed, implying that the amplitude of all beta bursts is reduced. Note that background levels of beta are shown as similar between conditions as our signal processing is focussed on burst behaviour. We cannot rule out additional changes in background levels of beta in the subthalamic nucleus, particularly during conventional DBS, but such changes are difficult to ascertain whilst amplifier noise floors may vary between conditions. aDBS = adaptive DBS; cDBS = conventional DBS.

Here we confirm that the subthalamic nucleus LFP in patients with Parkinson’s disease is characterized by bursts of beta activity of varying duration, and show that the amplitude of the beta bursts is reduced by both conventional and adaptive DBS. However, this is accomplished in different ways. In adaptive DBS it paradoxically occurs despite an increase in the frequency of bursts. However, in this condition there is a shift from long beta bursts to shorter bursts as long bursts are prematurely terminated by triggered stimulation. This both converts long bursts into shorter ones and frees up more time for spontaneously short bursts to occur. Critically, beta burst amplitude ramps up with burst duration, so that this shift in burst duration distribution with adaptive DBS explains the overall reduction in integrated burst amplitude in this stimulation condition. In contrast, in conventional DBS there is no change in the frequency of beta bursts and no change in the distribution of burst durations compared to the unstimulated state. By exclusion therefore, the reduction in integrated burst duration relative to the unstimulated condition must entail an attenuation of burst amplitude. This turns out to be equally distributed across short and long bursts. The contrasting effects of adaptive and conventional DBS are summarized in the schematic given in Fig. 7.

It is important to note that beta bursts were defined in DC corrected data, thereby removing any offset due to a general difference in beta amplitudes or amplifier noise floors between conditions and focusing on dynamic variations in beta amplitude. The difference in the incidence of short and long bursts between conditions therefore denotes a change in the distribution of burst durations. Nevertheless, the shift in burst duration distribution during adaptive DBS could have been due to a low beta amplitude (and hence poor signal-to-noise ratio) so that the bursts were predominantly driven by noise and accordingly shorter in duration. Fortunately, the conventional DBS state provides a control for this: the suppression of beta burst amplitude averaged across bursts of all durations was no different from that during adaptive DBS (Fig. 5B). Thus changes in power and signal-to-noise ratio are insufficient to explain the shift in burst duration distribution. Moreover, the distinct burst distribution during adaptive DBS relative to noStim and conventional DBS was strongly supported by the consistency of the findings across different amplitude thresholds. Key findings with respect to burst distribution were also reproduced by focusing on total time spent in short and long bursts, rather than just considering the number of bursts. In addition, the pattern in the dichotomized burst distribution was preserved when the broad band filtered signal (13–30 Hz) was considered instead of a signal narrowly filtered around the beta peak. The comparison between adaptive and random DBS, which was possible to conduct in eight hemispheres only (see ‘Materials and methods’ section), confirmed that the trimming of long beta bursts is specific to the adaptive DBS stimulation paradigm and not due to an intermittent delivery of stimulation *per se.*

### Significance of short beta bursts

Beta bursts are a well-established feature in the cortico-basal ganglia loop of healthy non-human primates (Murthy and Fetz, 1992, 1996; Feingold *et al.*, 2015). Beta amplitude also increases as burst duration increases in the healthy striatum, but the vast majority of beta bursts last no more than two to three cycles (Feingold *et al.*, 2015). Short duration beta bursts may therefore be physiological and functionally relevant, and in line with this, a higher proportion of shorter bursts was associated with less clinical impairment. The suggestion that shorter and hence weaker periods of synchronization in the beta band may be important in normal motor processing is in line with the evidence for such physiological activity at the cortical level (Murthy and Fetz, 1992, 1996; Kilner *et al.*, 2003) and the evidence pointing to task-dependent modulation of such activity within the basal ganglia (Courtemanche *et al.*, 2003; Feingold *et al.*, 2015). The increase in the frequency of short duration bursts during adaptive DBS may thus be one way in which adaptive DBS restores motor function, although it remains to be proven that such short bursts are physiological and functionally relevant in the human.

### Significance of long beta bursts

One of the most robust findings was the ramping up of the amplitude of LFP oscillations as beta burst duration increased. This implies the progressive synchronization of the neural elements summating to give the beta oscillations in the LFP and if unchecked, i.e. in long duration bursts, this leads to pervasive local synchronization in the beta band, with its attendant costs in terms of information coding capacity and ensemble performance (Brittain and Brown, 2014). In line with this, the frequency of long duration beta bursts in the subthalamic nucleus correlated with contralateral upper limb motor impairment in the absence of DBS. Conventional DBS also modulated long duration beta bursts, but not by changing their frequency, rather by attenuating their amplitude. However, this attenuation was relatively indiscriminate and affected short bursts as well. Indeed, this may provide a plausible explanation for the observation that conventional high frequency DBS can sometimes itself lead to paradoxical motor impairment under circumstances where more pathological longer duration bursts may be scarce or absent, such as in Parkinson’s disease patients with preserved motor performance off DBS at the time of testing (Chen, *et al.*, 2006*a*).

Nevertheless, long beta bursts were uncommon and, when defined as exceeding the 75th percentile amplitude, accounted for only ∼10% of the recorded signal duration. In this regard it is worth noting the evidence that elevated periods of beta activity within the motor system may have functional effects that outlast the elevation, at least in healthy humans (Gilbertson *et al.*, 2005; Androulidakis *et al.*, 2006; Lalo *et al.*, 2007).

### Clinical relevance of adaptive deep brain stimulation

The present findings link beta burst duration in the subthalamic nucleus, its modulation and motor performance. The correlation with motor performance revealed an interesting relationship between burst duration and clinical impairment. The proportion of long duration beta bursts was positively related to clinical impairment, while the proportion of short duration beta bursts was negatively related to clinical impairment in the unstimulated state. These results suggest that a long duration of uncontrolled synchronization has an important negative impact on motor performance, whereas we could speculate that short duration beta synchronization may impact positively (Eusebio and Brown, 2009; Brittain *et al.*, 2014). We therefore hypothesize that the selectivity and modulatory effect of adaptive DBS on long duration beta bursts might help explain the reported (but to be confirmed) wider therapeutic window of adaptive DBS over conventional DBS in treating motor symptoms. This could directly follow on from the shift in the distribution in beta burst duration or be secondary to the reduced time on stimulation and hence lower total electrical energy delivered, which then helps reduce side effects such as stimulation-induced speech impairment and dyskinesias (Little *et al.*, 2013, 2016; Rosa *et al.*, 2015).

### Adaptive deep brain stimulation in the future

The presented data do not serve as evidence of the clinical efficacy or efficiency of adaptive DBS, nor of any superiority of adaptive DBS over conventional DBS, or vice versa. This has to be established through further clinical trials. Likewise, the findings reported here do not preclude other innovative approaches to DBS, such as novel pulse parameters or coordinated reset (Kühn and Volkmann, 2016). However, the present findings suggest why adaptive and conventional DBS might potentially have different therapeutic performance, as they affect pathological networks in different ways. Moreover, they may help guide how adaptive DBS might be optimally delivered in the future. Key may be the selective and premature termination of longer beta bursts. The adaptive DBS system used to show clinical efficacy in two recent patient series (Little *et al.*, 2013, 2015) would leave bursts with a duration <∼500 ms untouched because of delays incurred by signal filtering and averaging, and stimulation ramping. However, the precise burst duration to be spared needs further investigation. Based on the present findings we suggest that an adaptive system that aims to shorten the duration of beta bursts should preferably have a high temporal resolution and a bang-bang (on/off regulation) control algorithm. A more gradual proportional–integral–derivative control policy with substantial signal smoothing might miss burst events and rather suppress beta amplitude across longer time courses. However, further studies are required to determine the most efficacious closed-loop control algorithm and then to compare the clinical performance of a closed-loop DBS system with the optimized control algorithm with that provided by established conventional DBS. Finally, long-term adaptive DBS is likely to need to self-optimize to allow for fluctuations in state over time. The present findings give more information about informative optimization targets, such as the matching of stimulation performance to an ideal distribution of burst durations or to achieve a given integrated beta burst amplitude.

### Limitations of this study

Bursts can be defined in many ways, and differences between stimulation conditions are illustrated for bursts exceeding a representative threshold equivalent to the 75th percentile of beta amplitude. Nevertheless, other thresholds were also tested and showed similar differences between stimulation conditions. Moreover, by concentrating on bursts of elevated beta activity we cannot comment on whether periods of lower beta amplitude contribute to function or dysfunction. Another limitation is that the relatively narrow pass band (3–37 Hz) of our amplifier and the presence of possible artefact at frequencies under ∼10 Hz prohibited the demonstration of any frequency selectivity to the differences in burst distributions. In addition it should be taken into account that the stimulation sessions took place only a few days after operation when stun effects are common (Chen, *et al.*, 2006*b*). Accordingly, our conclusions about the effects of different burst durations need to be validated in chronically implanted patients in whom stun effects have lapsed. Finally, it should also be noted that due to time constraints, we only tested selected items of the motor UPDRS related to patient fatigue, and organizational issues related to hospitalization. The role beta bursts play in those aspects of motor impairment not captured here, such as gait, remains to be investigated.

## Conclusion

Adaptive DBS may provide a means to relatively selectively regulate excessive beta synchronization by limiting beta burst duration, while leaving more shorter bursts of beta synchrony and beta burst-free periods unaffected. Whether this is translated into fewer side-effects than with conventional DBS under chronic conditions remains to be proven. However, the correlation between burst duration and clinical impairment suggests that the selectivity for long duration bursts might help explain the preliminary reports of the superior efficacy of adaptive DBS over conventional DBS in acute studies (Little *et al.*, 2013). There remains much to be explored about the clinical potential of adaptive DBS, but the present findings should help inform the design of optimal adaptive DBS systems for such trials.

## Supplementary Material

Supplementary Data

## Abbreviations

DBS: deep brain stimulation
LFP: local field potential
UPDRS: Unified Parkinson’s Disease Rating Scale
