# Supplementary material for: The modulatory effect of adaptive deep brain stimulation on beta bursts in Parkinson’s disease
Source: Brain. 2017 Feb 13;140(4):1053–67. doi: 10.1093/brain/awx010 (PMC5382944; doi:10.1093/brain/awx010)
Supplement: Supplementary Data [file awx010_supp.pdf]

Supplementary material

A

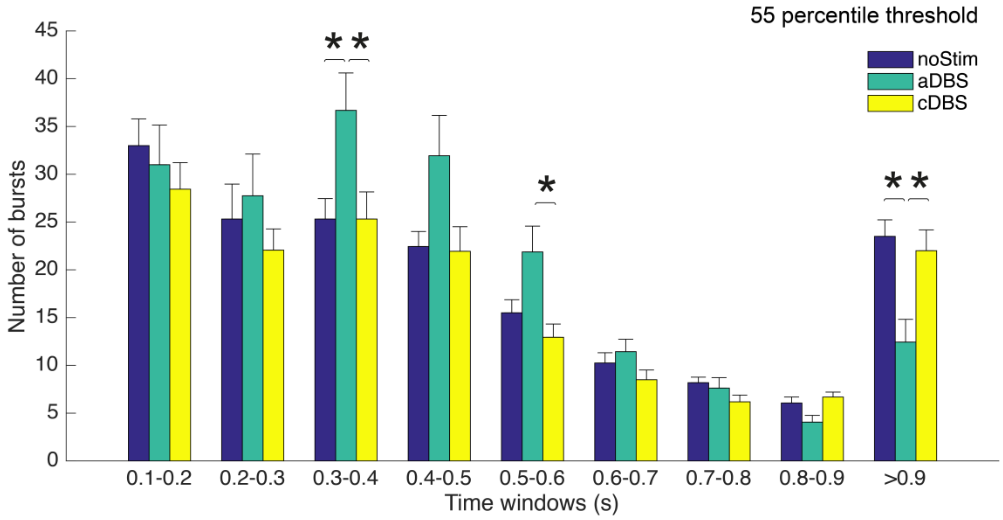

B

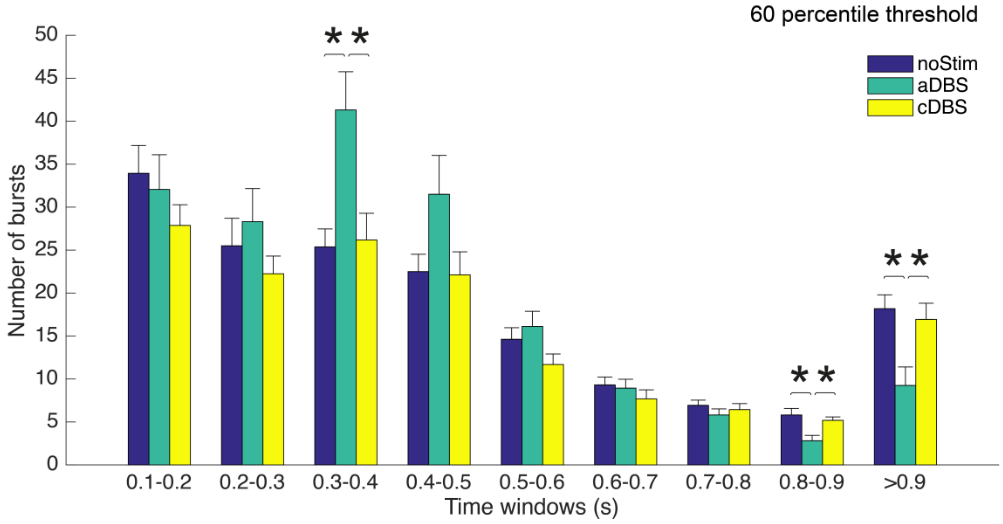

C

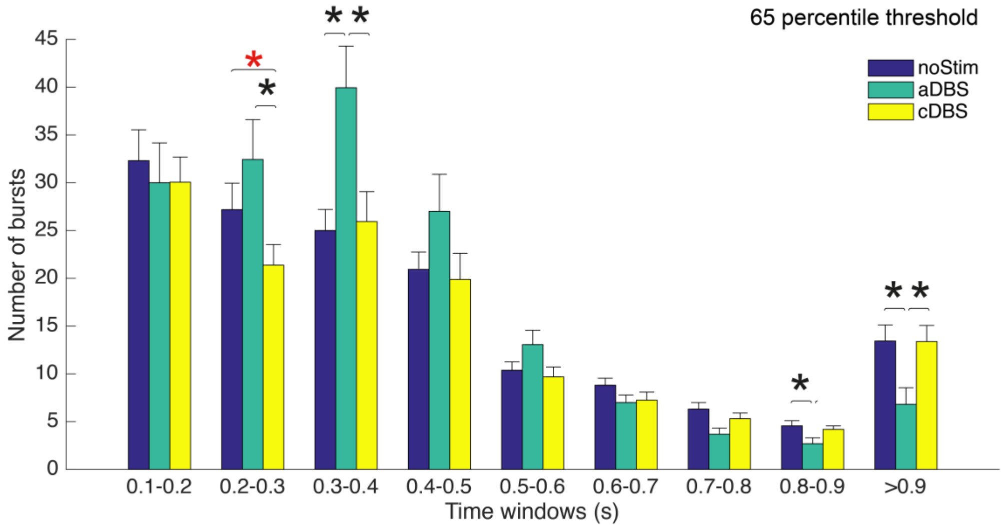

D

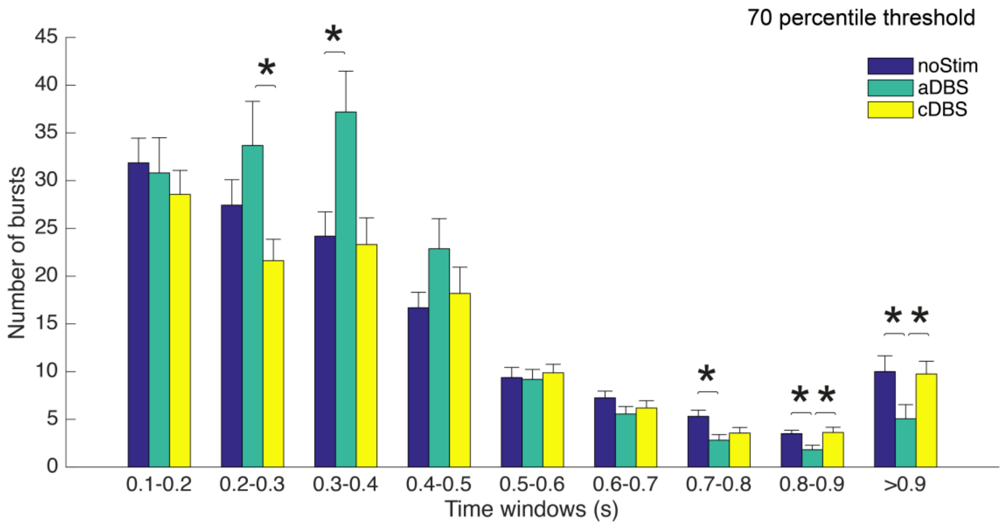
70 percentile threshold
noStim
aDBS
cDBS
Number of bursts
0
5
10
15
20
25
30
35
40
45
0.1-0.2
0.2-0.3
0.3-0.4
0.4-0.5
0.5-0.6
0.6-0.7
0.7-0.8
0.8-0.9
>0.9
Time windows (s)

E

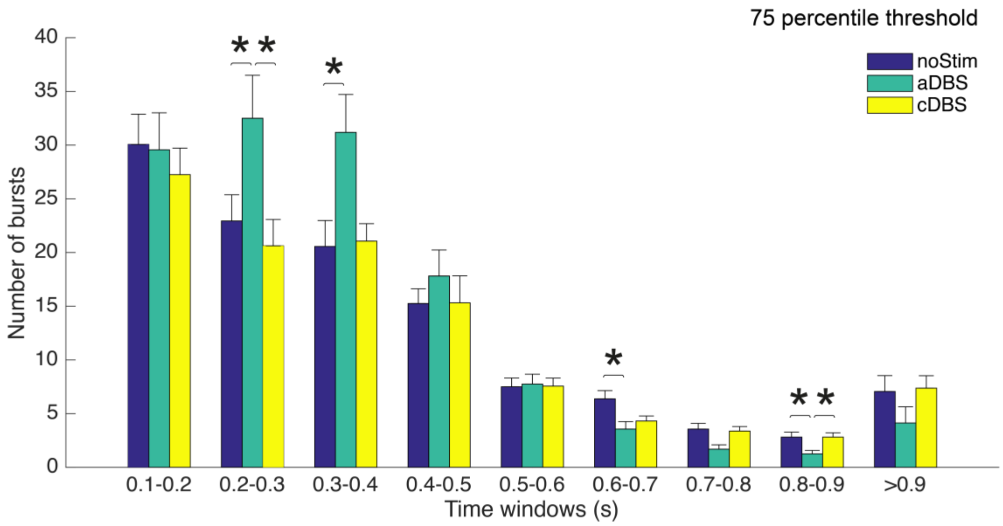
75 percentile threshold
noStim
aDBS
cDBS
Number of bursts
0
5
10
15
20
25
30
35
40
0.1-0.2
0.2-0.3
0.3-0.4
0.4-0.5
0.5-0.6
0.6-0.7
0.7-0.8
0.8-0.9
>0.9
Time windows (s)

F

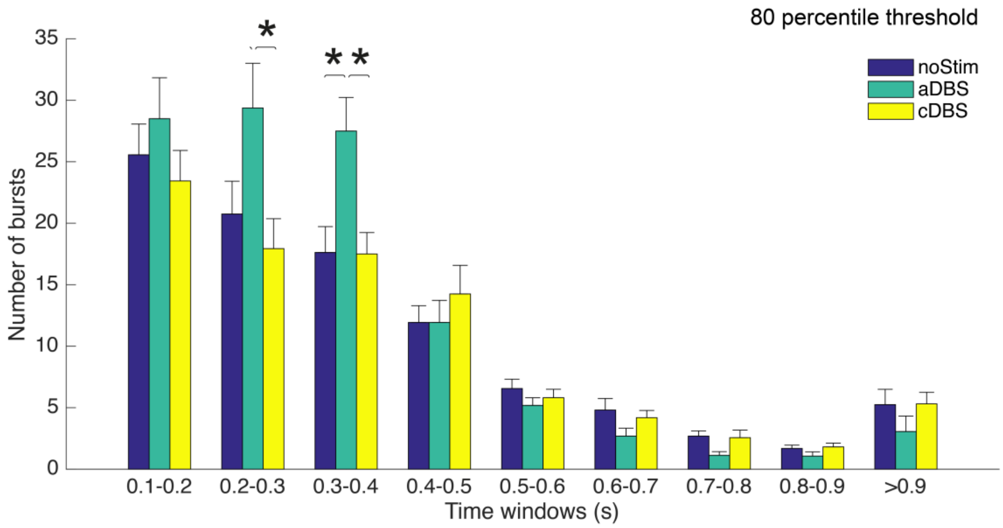
80 percentile threshold
noStim
aDBS
cDBS
Number of bursts
0
5
10
15
20
25
30
35
0.1-0.2
0.2-0.3
0.3-0.4
0.4-0.5
0.5-0.6
0.6-0.7
0.7-0.8
0.8-0.9
>0.9
Time windows (s)

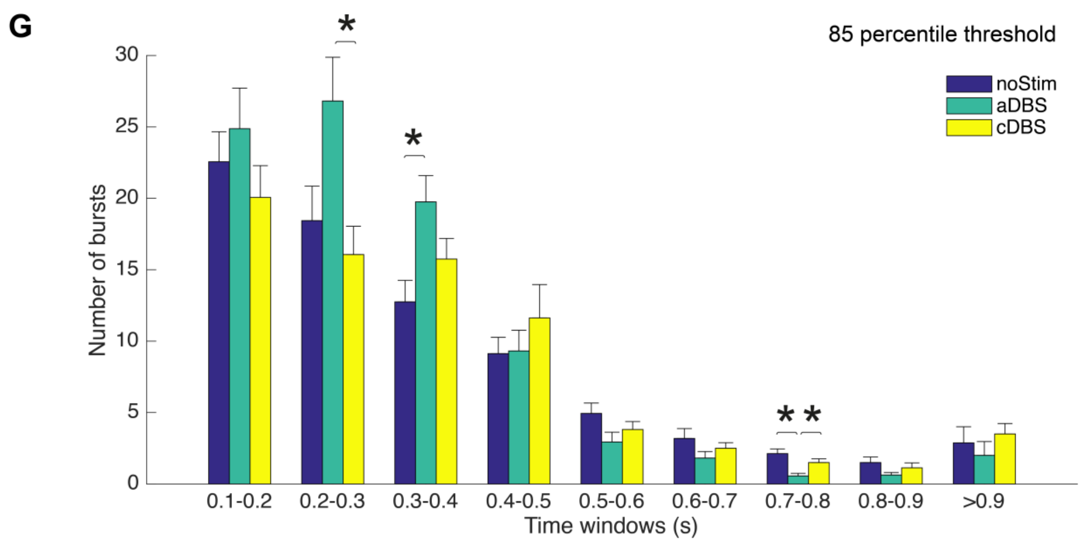

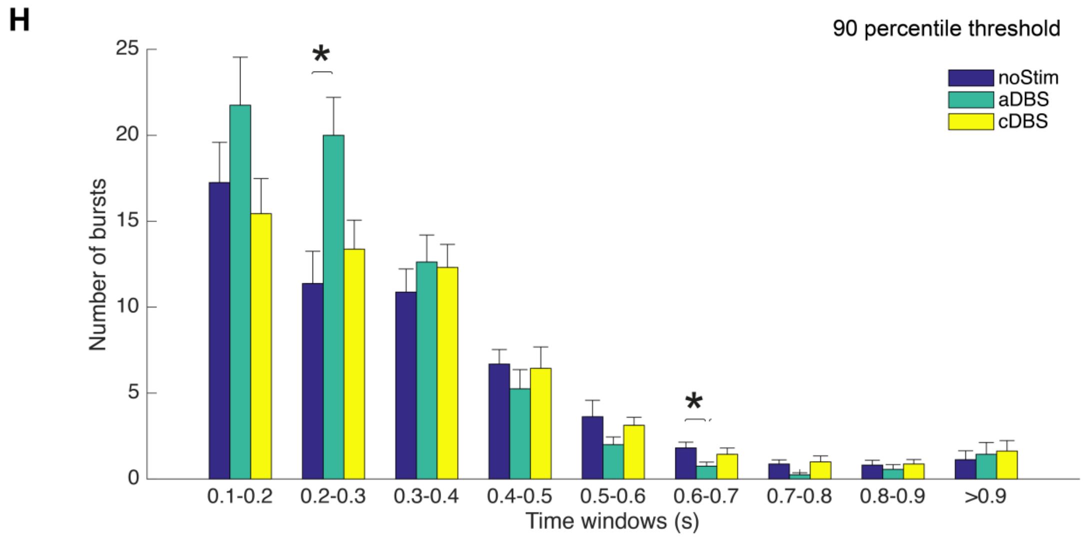

Supplementary figure 1: Beta burst distribution for burst definitions of different thresholds (A-H) during noStim, aDBS and cDBS. In comparison to noStim and cDBS, during aDBS there tended to be more bursts with shorter time windows/durations (<600ms) and less bursts with longer time windows (>600ms). Post-hoc tests were only carried out where the ANOVA for a given threshold was significant. * denotes post-hoc tests showing significant difference ($p<0.05$) between aDBS and cDBS or noStim, with the precise pairing denoted by the positioning of the asterix. The single instance of noStim significantly differing (exceeding) cDBS is denoted by *, and occurs with the 65th percentile threshold in the time window 200-300 ms. Otherwise there were no differences in the distribution of bursts of different duration between noStim and cDBS.

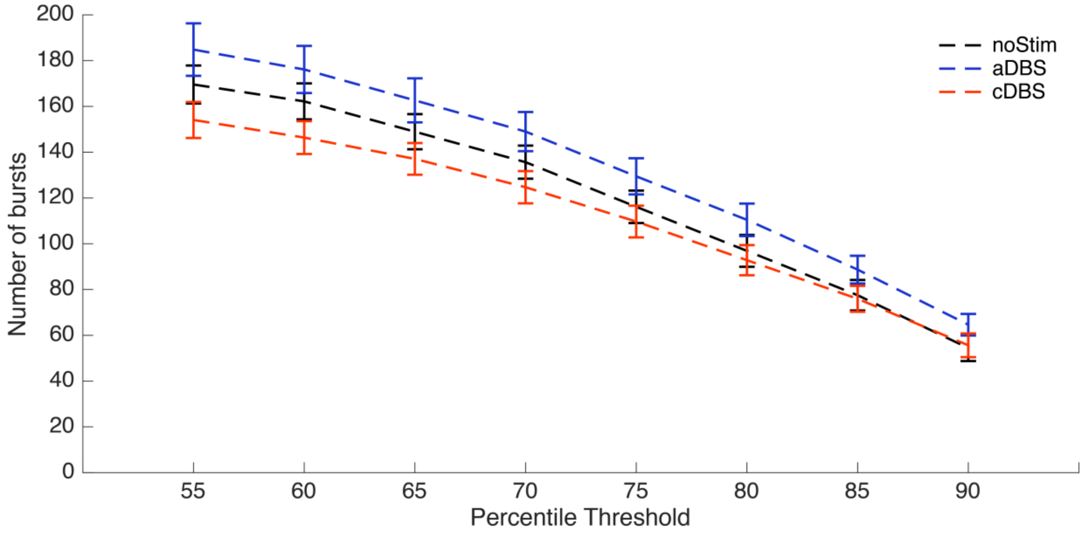

Supplementary figure 2: Total number of bursts across burst definitions of different thresholds. The total number of bursts for noStim, aDBS and cDBS shows a relatively uniform evolution across the different thresholds (55 percentile to 90 percentile). The RM-ANOVA (9 x 3 design) showed no significant main effect for the interaction between condition and threshold ($F_{(df\ 2.4,36.5)}$=1.371, p=0.268), despite the trend for aDBS having a higher total number of bursts than noStim and cDBS. This trend is most likely driven by the higher number of shorter bursts during aDBS.

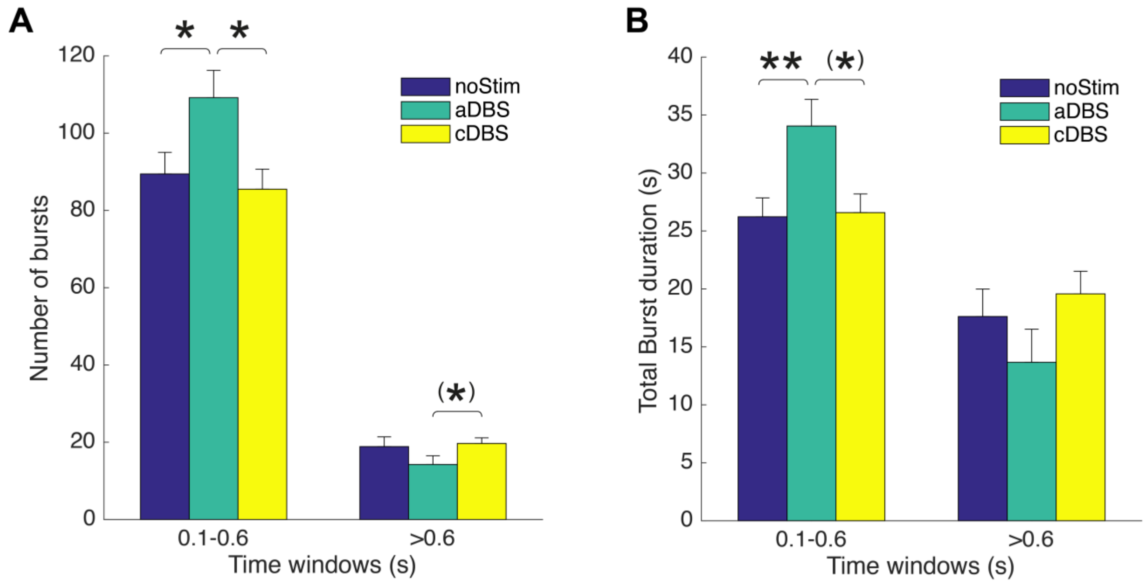

Supplementary figure 3: Burst distribution of broad band filtered (13-30 Hz) LFP signal. Data for the representative threshold (75 percentile) **(A)** illustrates the number of short and long bursts for noStim, aDBS and cDBS. The corresponding RM ANOVA confirmed a significant interaction between condition and burst duration ($F_{(df\ 1.4,20.9)}$=7.739, p=0.006). The post hoc analyses revealed a higher number of shorter bursts (100-600ms) in aDBS compared to noStim (t_{15}=3.190 p=0.018) and cDBS (t_{15}=2.894 p=0.033). An opposite trend was observed for longer bursts (>600ms) with a lower number of bursts in aDBS compared to cDBS (t_{15}=-2.240 p=0.122). No significant difference was found for longer bursts between aDBS and noStim (t_{15}=-1.113 p=0.850) and for short and long bursts between noStim and cDBS (t_{15}=0.677 p=1; t_{15}=-0.268 p=1). **(B)** A similar main effect in the RM ANOVA was found when the number of bursts was replaced with the total burst durations for short and long bursts ($F_{(df\ 1.3,19.9)}$=5.235, p=0.025). Post hoc analyses also revealed a longer total duration for shorter bursts (100-600ms) in aDBS compared to noStim (t_{15}=4.385 p=0.002) and the same trend was observed for cDBS (t_{15}=2.640 p=0.056) as well. No significant differences between aDBS and noStim/cDBS were found for long burst duration bursts (t_{15}=-0.884 p=1; t_{15}=-1.831 p=0.261). For short and long bursts there was no significant difference between noStim and cDBS (t_{15}=-0.180 p=1; t_{15}=-0.677 p=1). Values are represented as mean + SEM; *p<0.05, **p<0.01. Asterisks in brackets: p-value significant only before correction for multiple comparison.

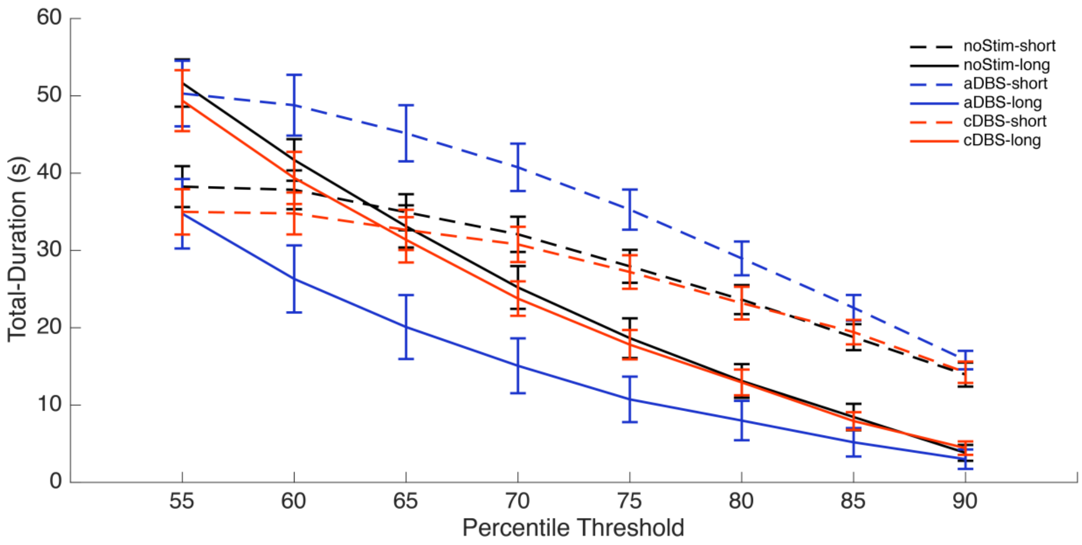

Supplementary figure 4: Total beta burst durations for short and long duration bursts across different thresholds in the three different conditions. This figure illustrates the consistent trend of aDBS spending more time in shorter bursts relative to noStim and cDBS, with an opposite trend when long bursts are considered. See results and figure 4D for the summarized statistics.

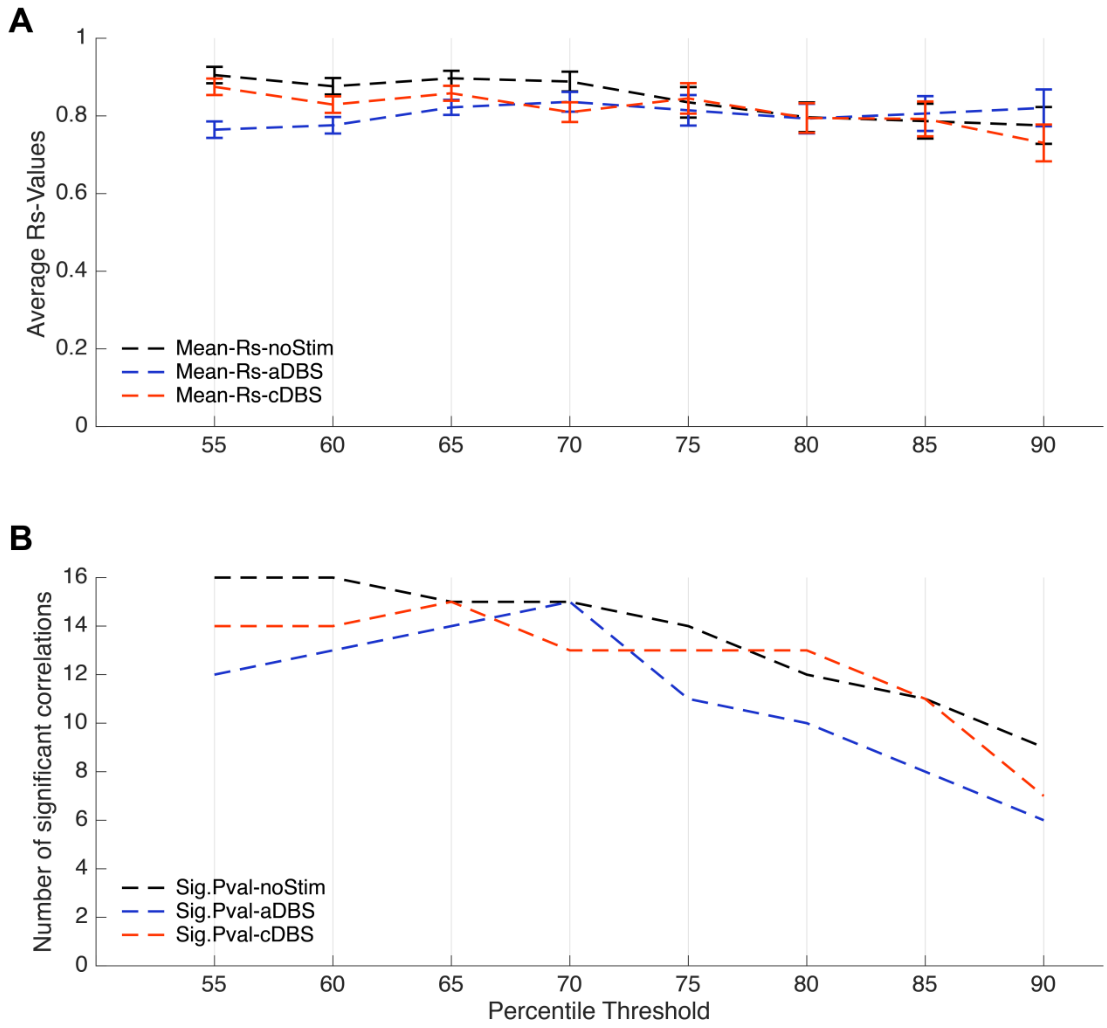

Supplementary figure 5: Relationship between burst duration and burst amplitude across the different thresholds. (A) illustrates the mean correlation coefficient (Spearman's rho) for the correlation between burst duration and burst amplitude for noStim, aDBS and cDBS. This shows a relatively constant relationship across the different thresholds. **(B)** shows the number of correlations with significant p-values (out of 16 hemispheres) for noStim, aDBS and cDBS. These tend to fall off at higher thresholds as the number of bursts falls.

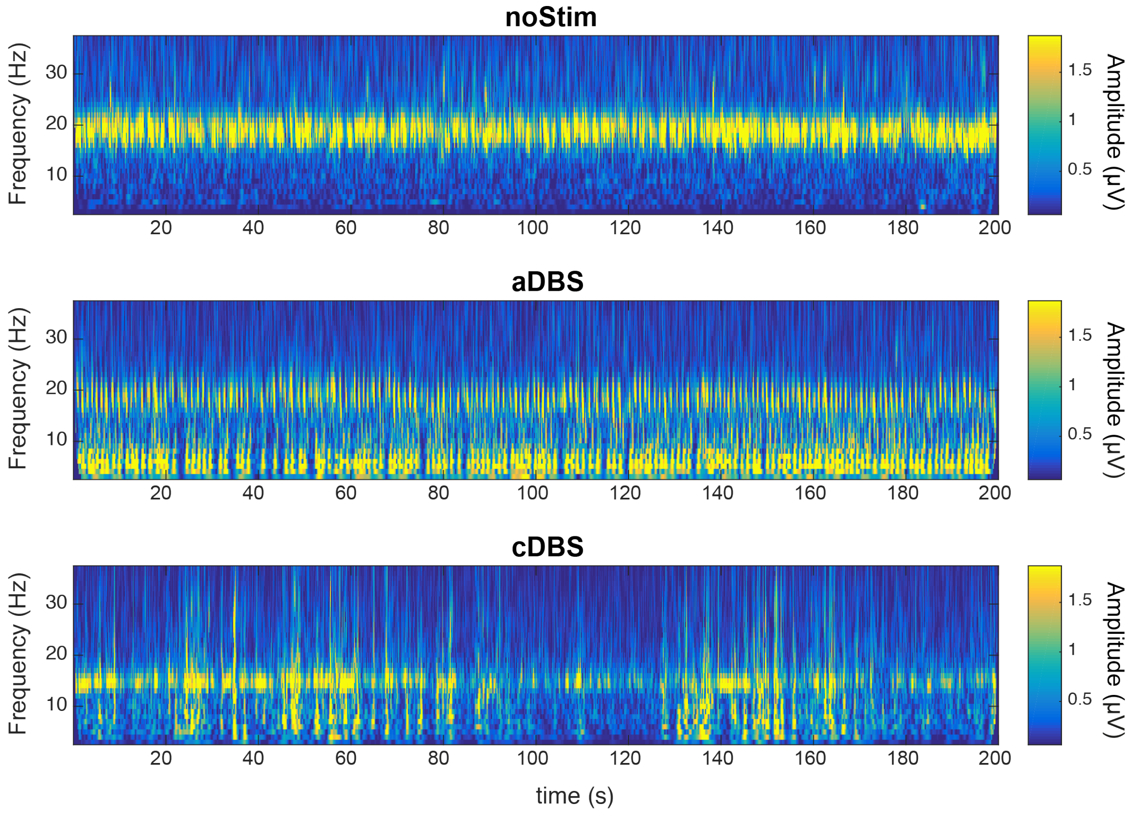

Supplementary figure 6: Time frequency spectra. Time frequency spectra over 3-37 Hz for the whole recording segment (200s) for noStim, aDBS and cDBS in one patient. Clear beta activity can be delineated, which is stronger during noStim compared to aDBS and CDBS. Note the power at frequencies under 10 Hz, which is more marked during aDBS (case 7, right side).

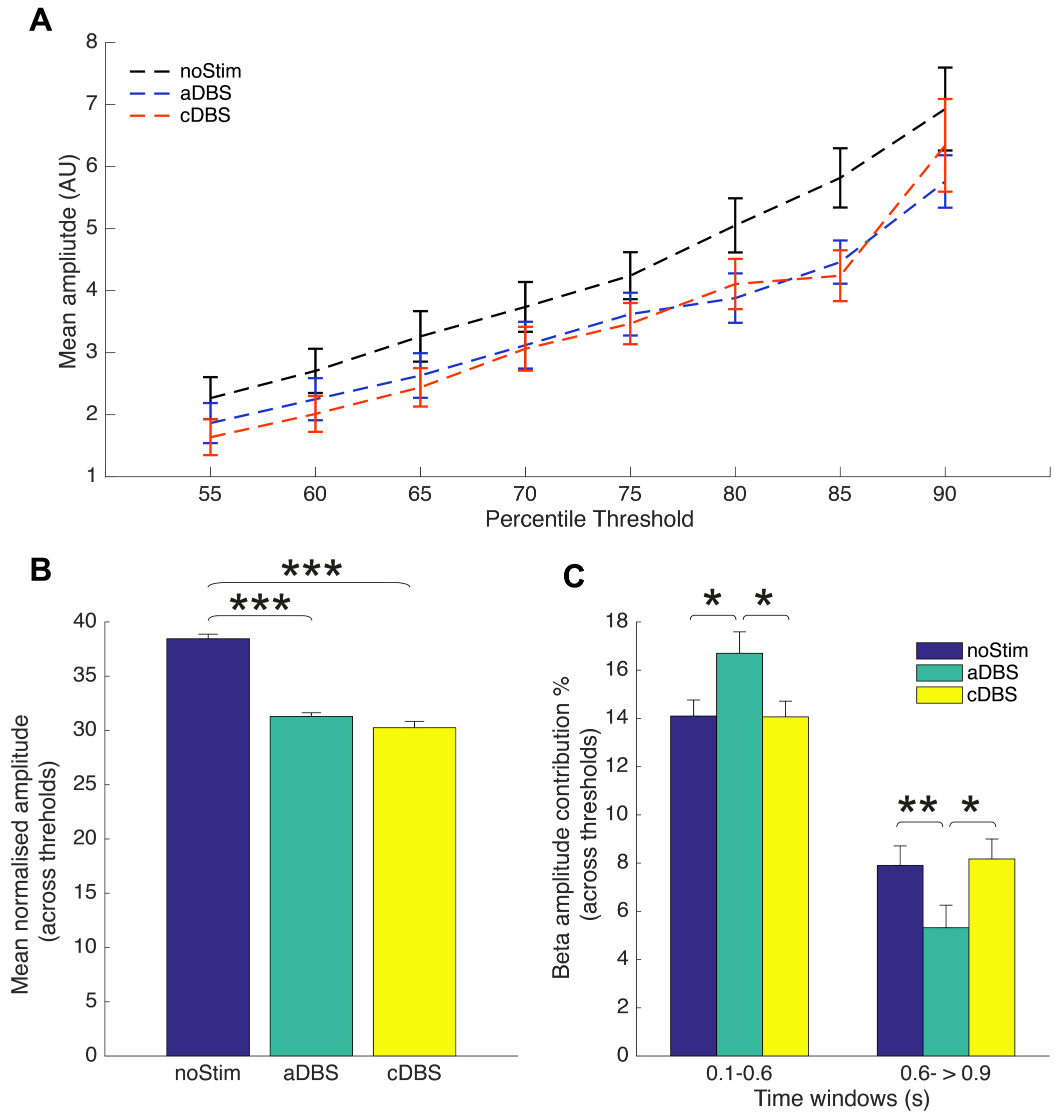

Supplementary Figure 7: Mean and integrated normalised burst amplitudes across different thresholds. (A) shows the mean amplitude for all the three conditions across the different thresholds. Mean burst amplitude during NoStim was higher than in aDBS and cDBS, as confirmed in **(B)**. RM-ANOVA across thresholds shows a positive main effect of stimulation ($F_{(df\ 2,14)}$=63.274, p<0.001) and the post hoc tests confirms a significantly higher burst amplitude in noStim compared to aDBS (t_6=14.570, p<0.001) and cDBS (t_6=8.552, p<0.001). No difference was found between aDBS and cDBS (t_6=1.216, p=0.790). **(C)** Illustrates the integrated amplitude (normalised to 100%, which corresponds to total integrated amplitude summed across all the time windows) for short bursts (100-600ms) and long bursts (>600ms) and averaged across thresholds. RM-ANOVA across thresholds showed a significant main effect for the interaction of condition and duration ($F_{(df\ 1.4,30)}$=10.310, p=0.002). Post hoc tests showed that the percentage of integrated amplitude of short bursts was higher in aDBS compared to noStim (t_{15}=3.459, p=0.011) and cDBS (t_{15}=3.055, p=0.024). The opposite was true for longer bursts, where the percentage of integrated amplitude from longer bursts in noStim and cDBS was higher than in aDBS (t_{15}=-3.826, p=0.005; t_{15}=-3.392, p=0.012). No difference was found between noStim and cDBS, either for short bursts (t_{15}=0.077, p=1) nor for longer bursts (t_{15}=-0.593, p=1). Values are represented as mean+SEM; *p<0.05, **p<0.01, ***p<0.001.

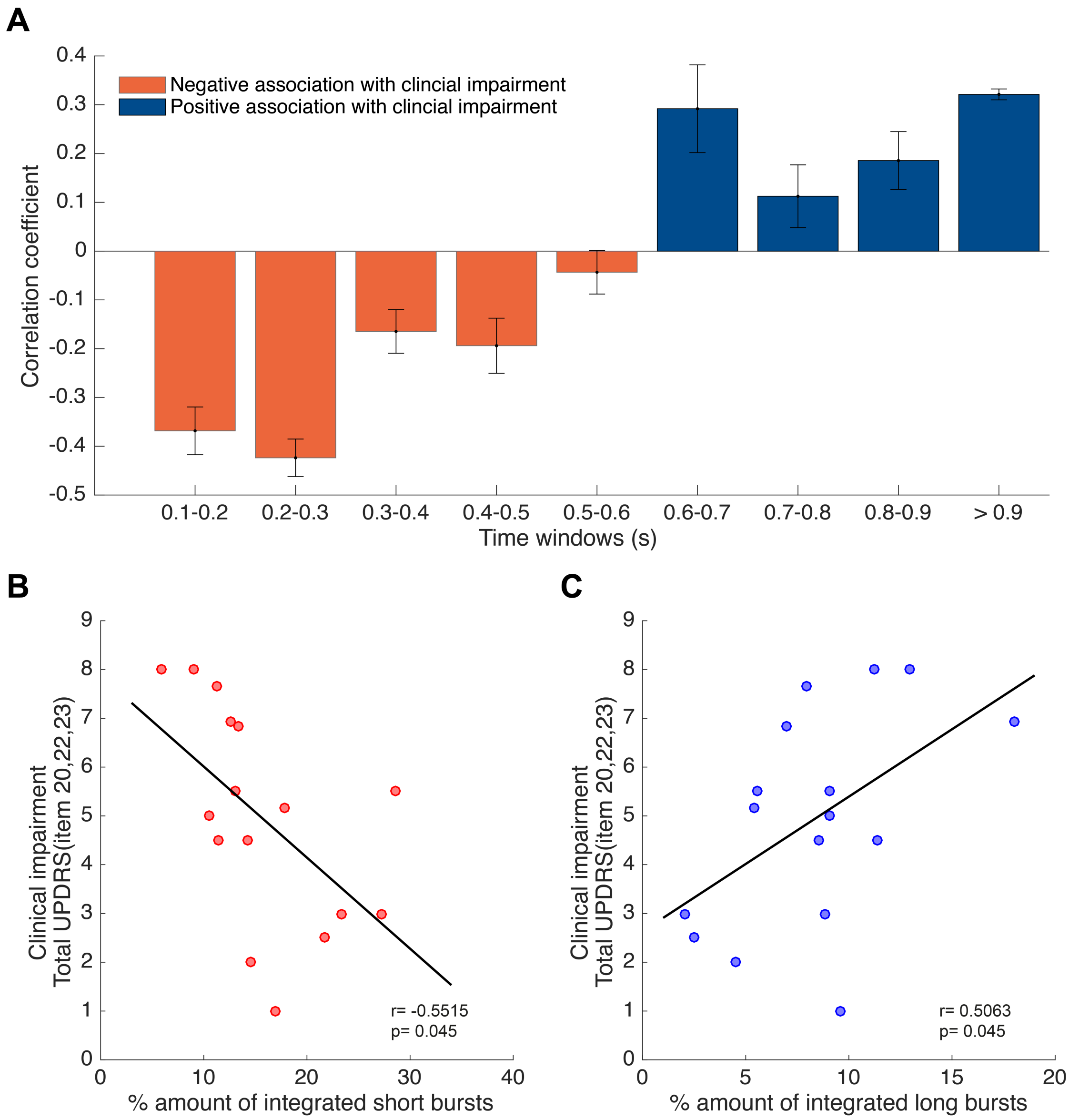

Supplementary Figure 8: Clinical correlation between integrated bursts and clinical impairment. (A) Pearson's correlations between clinical impairment and integrated burst amplitude (normalised to 100%, which corresponds to total integrated amplitude summed across all time windows) during different burst time windows across the various thresholds. These show that shorter bursts tend to be negatively correlated with clinical impairment and longer bursts tend to be positively correlated with clinical impairment. **(B)** Example scatter plot of percentage amount of short bursts (of 200-300ms duration) and clinical impairment (UPDRS Part III items 20, 22 and 23 contralateral to the recording side). **(C)** Example scatter plot of percentage amount of long bursts (700-900ms) and clinical impairment. B and C are data for representative threshold (75 percentile).
